# Screening for refractive error among primary school children in Bayelsa state, Nigeria

**DOI:** 10.11604/pamj.2013.14.74.1345

**Published:** 2013-02-25

**Authors:** Ibeinmo Opubiri, Chinyere Pedro-Egbe

**Affiliations:** 1 Department of Ophthalmology, Niger Delta University Teaching Hospital, Bayelsa State; 2 Department of Ophthalmology, University of Port Harcourt teaching Hospital, Rivers State

**Keywords:** Refractive error, Screening, School children

## Abstract

**Introduction:**

Vision screening study in primary school children has not been done in Bayelsa State. This study aims to screen for refractive error among primary school children in Bayelsa State and use the data to plan for school Eye Health Program.

**Methods:**

A cross sectional study on screening for refractive error in school children was carried out in Yenagoa Local Government Area of Bayelsa State in June 2009. A multistage sampling technique was used to select the study population (pupils aged between 5-15 years). Visual acuity (VA) for each eye, was assessed outside the classroom at a distance of 6 meters. Those with VA ≤6/9 were presented with a pinhole and the test repeated. Funduscopy was done inside a poorly lit classroom. An improvement of the VA with pinhole was considered refractive error. Data was analyzed with EPI INFO version 6.

**Results:**

A total of 1,242 school children consisting of 658 females and 584 males were examined.About 97.7% of pupils had normal VA (VA of 6/6) while 56 eyes had VAs ≤ 6/9. Of these 56 eyes, the visual acuity in 49 eyes (87.5%) improved with pinhole. Twenty seven pupils had refractive error, giving a prevalence of 2.2%. Refractive error involved both eyes in 22 pupils (81.5%) and the 8-10 years age range had the highest proportion (40.7%) of cases of refractive error followed by the 9-13 year-old age range (37%).

**Conclusion:**

The prevalence of refractive error was 2.2% and most eyes (97.7%) had normal vision.

## Introduction

Refractive error is an optical defect intrinsic to the eye which prevents the light from being brought to a single focus on the retina thus reducing normal vision [1]. Refractive error is a major contributor to visual impairment which is a significant cause of morbidity in children worldwide [2]. Since children do not usually complain of visual difficulties, early detection and prompt treatment of eye disease is important to prevent vision problems and eye morbidities which could affect their learning ability, personality and adjustment in school [3, 4].

Screening is the search for unrecognized disease or defect by means of rapidly applied test, examinations or other procedures in apparently healthy individuals [5]. A screening test is not intended to be a diagnostic test, it is only an initial examination. Those who are found to have positive test results are referred to an ophthalmologist for further diagnostic work-up and treatment [5].

A study on vision screening for refractive error among school children is yet to be carried out in Bayelsa State. In a study [6] on vision screening in primary school children in Enugu Nigeria, the prevalence of refractive error was 7.4%. Faderin [7] in her study on refractive error in pupils of Army children primary school in Lagos Nigeria found a prevalence of 7.3%. A similar study [8] on vision survey of school children in a rural community in south-east Nigeria observed a lower refractive error prevalence of 4.2%.

Kawuma and Mayeku [9] in Uganda found the prevalence of refractive errors in primary school children to be 11.6%. The high prevalence in the study may be partly due to the relatively small study population of 623 pupils. The Kawuma study contrasted with a similar study by Wedner [10] in rural Tanzania which showed a low prevalence of 1% for refractive error in school children aged 7-19 years. The lower prevalence in the Wedner [10] study is likely to be due to the reason that only the proportion of pupils with a visual acuity of less than 6/12 were considered in the study.

Padhye et al [11] in their study of the prevalence of uncorrected refractive error among urban and rural school children in Maharashtra, India, observed that the prevalence of uncorrected refractive error was higher in urban school children.

The Nigerian operational plan for the implementation of the vision 2020; [12] The Right to Sight document (2007-2011), proposed the establishment of school eye health screening in each Local Government Area to identify all cases of visual impairment.Early detection of a vision problem can have educational, behavioural and certainly, quality of life benefits. [13]

### Background information on study area

Yenagoa Local Government Area (LGA) is in Bayelsa State, Nigeria. Yenagoa LGA is made up of upland and riverine communities connected by tarred roads and bridges. The main occupation in the rural areas is farming and fishing, while in the city there are more of civil servants and traders. The people are predominantly of the Ijaw and Epie-Atissa ethnic nationality.

According to records in the Bayelsa State Ministry of Education there are 68 public primary schools and 18 approved private primary schools in Yenagoa LGA with a combined estimated student population of 35,802 pupils. Records from the Yenagoa LGA Basic Education Authority (LGBEA), shows that the area is divided into 3 education zones: Epie/Atissa, Gbarain/Ekpetiama and Biseni/Okordia.

There is currently no existing school health services program in Yenagoa Local Government Area. This study is expected to provide relevant data for the effective planning of School Eye Health Programs for the State.

## Methods

This study was conducted on primary school children in Yenegoa Local Government Area of Bayelsa State, Nigeria. Children in primary basic 1 to 6 and aged 5 to 15 years were included in the study. The United Nations children fund (UNICEF) definition of childhood as a period of life before 16 years of age was used. Children in Special Schools for the blind in the Yenegoa LGA were excluded from the study.

A multi-stage sampling technique was used to select pupils in the study population. Stage 1 sampling involved the selection of 3 clusters based on the three Educational Zones in the LGA. The second stage sampling involved stratification of schools into public and private, and in stage 3, five schools were selected (4 public and 1 private) based on the ratio of schools in the clusters and the population of pupils in the schools. The schools were randomly selected from a sampling frame of schools in each zone (public separated from private). All pupils in the selected schools were included in the study. The minimum sample size was 1,123.

Ocular examination included visual acuity (VA) with and without pinhole and funduscopy. VA was assessed outside at a distance of 6 meters while funduscopy was done inside a poorly lit part of the classroom. VA was determined separately for each eye and where it was 4 ≤ 6/9, a pinhole was presented and the test repeated. An improvement of the VA with pinhole was considered refractive error and a VA of ≤ 6/12 was regarded as reduced vision. A pilot study was carried out three days to the study in a primary school not in the study sample.

Ethical clearance was obtained from the Ethics Committee of the University of Port Harcourt Teaching Hospital and written consent from the Bayelsa State Ministry of Education and the Yenegoa Local Government Basic Education Authority. Personal data were recorded in a predesigned and pretested questionnaire and analysed using the Epidemiological information software - EPI INFO version 6.

## Results

There were 1,295 pupils in the class registers of the selected primary schools 42 pupils (3.2%) were absent from school on the screening days, giving a coverage rate of 96.8%. Of the 1,253 pupils screened, 11 were excluded from the study as they were at least ≥16 years. The primary 5 class, with 228 (18.3%) pupils had the highest number of pupils per class. Of the 1,242 pupils examined, 1,043 (84%) were from public schools and 199 (16%) from private schools, giving a public to private pupil ratio of 5:1.Those aged 8-13 years made up 77.8% of the study population (Table 1). There were 658 females and 584 males, giving a slight female preponderance ratio of 1.1:1.

**Table 1:** Age and Sex Distribution of the study population

Age range	Male	Female	Total	Percentage
**5–7**	122	121	233	18.7
**8–10**	254	251	505	40.7
**11–13**	195	266	461	37.1
**14-15**	23	20	43	3.1
**Total**	**584**	**658**	**1242**	**100%**

A total of 1,242 school children consisting of 658 (53%) females and 584 (47%) males were examined.About 97.7% normal VA (VA of 6/6) while 56 eyes had VAs ≤ 6/9. Of these 56 eyes, the visual acuity in 49 eyes (87.5%) improved with pinhole. Twenty six eyes (1%) had visual acuity of ≤6/12 and 22 (88%) improved with pin-hole. A total of 27 pupils had refractive error, giving a prevalence of 2.2%. Refractive error involved both eyes in 22 pupils (81.5%) and one eye in 5 pupils (18.5%). Table 2 shows that, the 8-10 year age-range had the highest proportion (40.7%) of cases of refractive error followed by the 9-13 year age group (37%) and that both sexes were almost equally affected.

**Table 2:** Age and sex distribution of refractive errors

AGE RANGE	SEX		TOTAL	FREQUENCY%
	M	F		
5–7	1	2	3	11.1
8–10	7	4	11	40.7
11–13	5	5	10	37.0
14–15	1	2	3	11.1
**Total**	**14**	**13**	**27**	**100**

About 97.7% of the pupils had normal vision (VA= 6/6). Fifty six eyes (2.2%) had visual acuity ≤ 6/9 while 26 eyes (1.0%) had visual acuity of 6/12 or worse. Two pupils (0.1%) had visual acuity of <6/18 in the better or worse eye (Table 3). One pupil (0.05%) with visual acuity of Hand Motion was the only case of monocular blindness recorded in the study.

**Table 3:** Unaided visual acuity in study population

Eyes	6/6	6/9	6/12	6/18	6/24	HM	Total
**Right**	1212(48.8%)	15(0.6%)	9(0.4%)	5(0.2%)	1(0.05%)	0	1242
**Left**	1215(48.9%)	16(0.6%)	6(0.2%)	4(0.2%)	0	1(0.05%)	1242
**Total**	2427(97.7%)	31(1.2%)	15(0.5%)	9(0.4%)	1(0.05%)	1(0.05%)	2484

* Hand Motion (HM)

Fifty six eyes with visual acuity of 6/9 or less where presented with pinhole. Of these, 87.5% improved with pinhole. Twenty two (88%), of the 26 eyes with visual acuity ≤ 6/12, improved when presented with pinhole (Table 4).

**Table 4:** Visual Acuity with Pin Hole

Eyes	6/6	6/9	6/12	6/18	NI	Total
Right	22(37.5%)	3(5.3%)	1(1.8%)	1(1.8%)	3(5.3%)	29
Left	21(39.2%)	1(1.8%)	0	0	4(7.1%)	27
**Total**	**43(76.7%)**	**4(7.1%)**	**1(1.8%)**	**1(1.8%)**	**7(12.4%)**	**56**

NI: no improvement

## Discussion

The visual experience of a child plays a significant role in his/her psychological, physical and intellectual development [13]. Visual impairment due to refractive error is a significant cause of morbidity in children worldwide [14, 15]. Many of the causes of visual impairment in childhood are avoidable, being either preventable or treatable [15]. There is therefore, a greater urgency when dealing with children who have visual impairment, as delay in treatment can lead to amblyopia [16].

The prevalence of refractive error in this study was 2.2%. This prevalence of refractive error falls within the WHO prevalence of 2-10% worldwide [10]. It is also similar to the report of Chuka-Okosa et al [17] in Enugu, South-Eastern Nigeria but much lower than the study of Balogun [18] in Lagos Island. Incidentally, Balogun's study involved pupils from both public and private primary schools just like this study and the reason for the wide disparity in results is not immediately apparent because Lagos is a more cosmopolitan city than Yenegoa and one would have expected a much lower result than that reported in our study.

On the other hand, Ajaiyeoba [19] in his study on blindness and visual impairment among school children in a rural community in South Western Nigeria, observed a lower refractive error prevalence of 0.87%. Ajaiyeoba's result compared to ours is not unexpected as the South-Westerners are more highly educated than those in Bayelsa State where our study took place. The studies by Nkanga [6] in Enugu and Faderin [7] in Lagos, both in cosmopolitan cities, also observed high prevalence of refractive error ((7.4% and 7.3% respectively). The variation in the prevalence of refractive error in these regions may be related to ethnic differences and in the case of Lagos and Enugu, the large heterogeneous population. The differences in the prevalence of refractive error in these studies, may also in part, be related to the methodologies used in identifying pupils with refractive error. In this study 97.7% of eyes had normal vision while those with visual acuity of 6/9 or better were 98.9%. Ugochuchukwu [8] in his study South-Eastern Nigeria got similar result.

The prevalence of pupils with an uncorrected visual acuity of 6/12 or less in this study was 1%. The refractive error study in children (RESC) carried out in Durban South Africa, involving children 5-15 years of age, found a prevalence of uncorrected visual acuity (VA of 6/12 or less) of 1.4%, decreasing to 0.32% with correction [20]. A similar study (RESC) in Nepal [21] had a prevalence of 2.9% of uncorrected visual acuity of 6/12 or less. In the study (RESC) in China, Zhao [22] found a high prevalence of uncorrected visual acuity of 6/12 or less, in at least one eye to be 12.8% and this decreased to 1.8% after correcting for refractive error.

Refractive error was responsible for most of the cases of reduced vision (84.6%) in this study. The prevalence of visual impairment in this study was 0.1% with refractive error making up 50% of this. A similar finding was observed by Ajaiyeoba [19], where refractive error accounted for 58% of visual impairment. The study by Ugochuku [8] showed a lower prevalence of visual impairment, with refractive error accounting for 33% of cases.

It was observed in this study that materials (visual acuity chart, vision recording book and other items) given to trained schoolteachers, were not being used but firmly locked up as “souvenirs” in the head teachers office, a situation that will not allow for sustenance of the program.

## Conclusion

The prevalence of refractive error in this study was 2.2%.Vision screening should routinely be done at school entry, midway through school and at completion of primary school, for early detection and treatment of eye diseases. The School Eye Health Screening Program should however, be strengthened to provide among other things, spectacles for students to correct refractive errors, after proper ophthalmological assessment.
